# An Altered Metabolism in Leukocytes Showing *in vitro* igG Memory From SARS-CoV-2-Infected Patients

**DOI:** 10.3389/fmolb.2022.894207

**Published:** 2022-06-30

**Authors:** G. Fanelli, F. Gevi, G. Zarletti, M. Tiberi, V. De Molfetta, G. Scapigliati, A. M. Timperio

**Affiliations:** ^1^ Department of Ecological and Biological Sciences, University of Tuscia, Viterbo, Italy; ^2^ Department of Innovativative Biology, Agro-food, and Forestry, University of Tuscia, Viterbo, Italy

**Keywords:** metabolomics, COVID-19, *in vitro* B-cell memory, cell-ELISA, mass spectrometry

## Abstract

Coronavirus disease 2019 (COVID 19) is a systemic infection that exerts a significant impact on cell metabolism. In this study we performed metabolomic profiling of 41 *in vitro* cultures of peripheral blood mononuclear cells (PBMC), 17 of which displayed IgG memory for spike-S1 antigen 60–90 days after infection. By using mass spectrometry analysis, a significant up-regulation of S-adenosyl-Homocysteine, Sarcosine and Arginine was found in leukocytes showing IgG memory. These metabolites are known to be involved in physiological recovery from viral infections and immune activities, and our findings might represent a novel and easy measure that could be of help in understanding SARS-Cov-2 effects on leukocytes.

## Introduction

The SARS-CoV-2 virus induces an unprecedented pandemic (COVID-19) characterized by a range of respiratory symptoms that may progress to acute respiratory distress syndrome (ARDS), and multi-organ dysfunction (Zhou et al., 2020). SARS-CoV-2 triggers both innate and specific immune responses, and once the virus gains access to the target cell, the host’s immune system mainly recognizes its surface epitopes including the spike-S1 protein (Shen et al., 2020). Among other effects, a recent study showed that COVID-19 leads to a reduced population of regulatory lymphocytes, inducing an increase in inflammatory responses, cytokine production, and proceeds toward tissue damage and systemic deficiency of organs (Shah et al., 2020). However, there is very limited understanding of the immune responses, especially adaptive immune responses to SARS-CoV-2 infection. To this regard Ni, Ling et al., characterized SARS-CoV-2-specific humoral and cellular immunity in recovered patients funding that both B and T cells participate in immune-mediated protection against viral infection (Ni et al., 2020). In addition, Turner JS et al., conducted a study among recovered individuals who experienced mild SARS-CoV-2 infection. The authors reported that mild infection induced a robust antigen-specific, long-lived humoral immune memory in humans (Turner et al., 2020). Although longer follow-up studies are needed, the first evidence confirms the protective effect of recovery from the previous infection. Hence, it is conceivable to speculate that at later stages after infection, patients that recovered from the pathology developed an immune response with memory and maintained antiviral defense, however the cellular physiological pathways underlying these mechanisms remain to be better understood. To this, a piece of knowledge on cellular processes that could be related to an infection can be achieved through metabolomics, an emerging tool applied to systems immunology platforms and already employed in COVID-19 studies (Diray-Arce et al., 2020). This preliminary study aimed to investigate possible relationships between leukocytes displaying an IgG antibody memory to SARS-CoV-2 (Zarletti et al., 2020) and their metabolic profile, to detect possible metabolites involved in the late stages of antiviral defenses.

## Results

**FIGURE 1:**
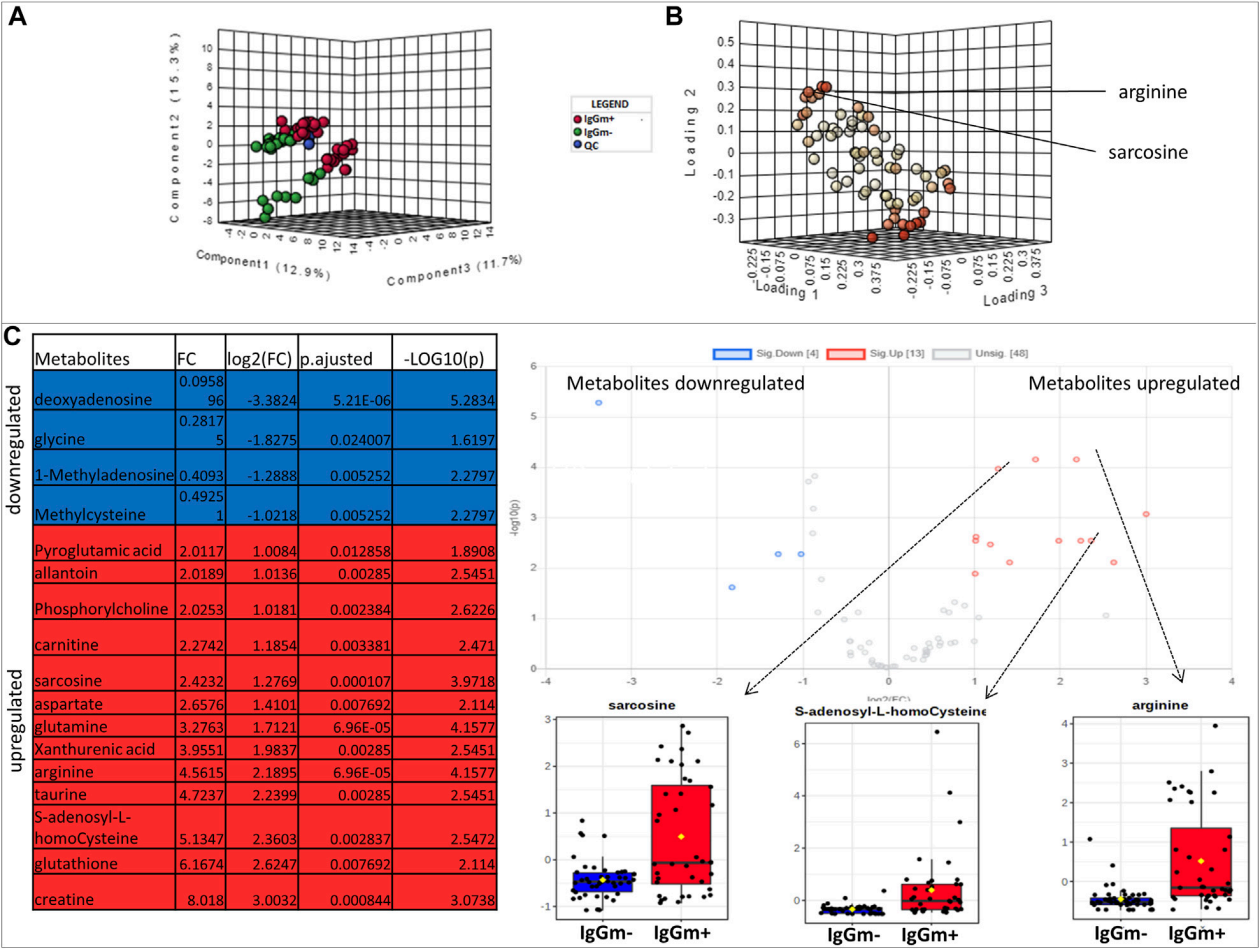
**(A)** Multivariate statistical analysis based on untargeted metabolite profile data derived from leukocytes of patients IgGm+ (Red), IgGm- (green) and QC (Blue) **(B)** shows the loading plot of the metabolites in the analyzed samples projected into the PLS-DA model. The loading plot is used to assess the features that have the greatest influence on each component and red spots represent metabolites that have strong contributions to the separation in the loading plot **(C)** Volcano plot showing the most significant metabolites found by univariate analysis. The table represents the results of volcano plots: for volcano plot analysis the conditions of fold change were ≥1.5 (*x*-axis) and the false discovery rate-adjusted *p*-value threshold was set at ≤ 0.05 (*y*-axis). The levels of metabolites were significantly different in the IgGm + compared to IgGm- The volcano plot summarizes both fold-change and *t*-test criteria for all metabolites. The blue-highlighted table shows metabolites up-regulated in IgGm-leukocytes, whereas in the red-highlighted metabolites are up-regulated in IgGm + leukocytes. The more significantly altered metabolites in IgGm + leukocytes are displayed as bar plots and were sarcosine, s-adenosyl-homocysteine, and arginine.

**FIGURE 2:**
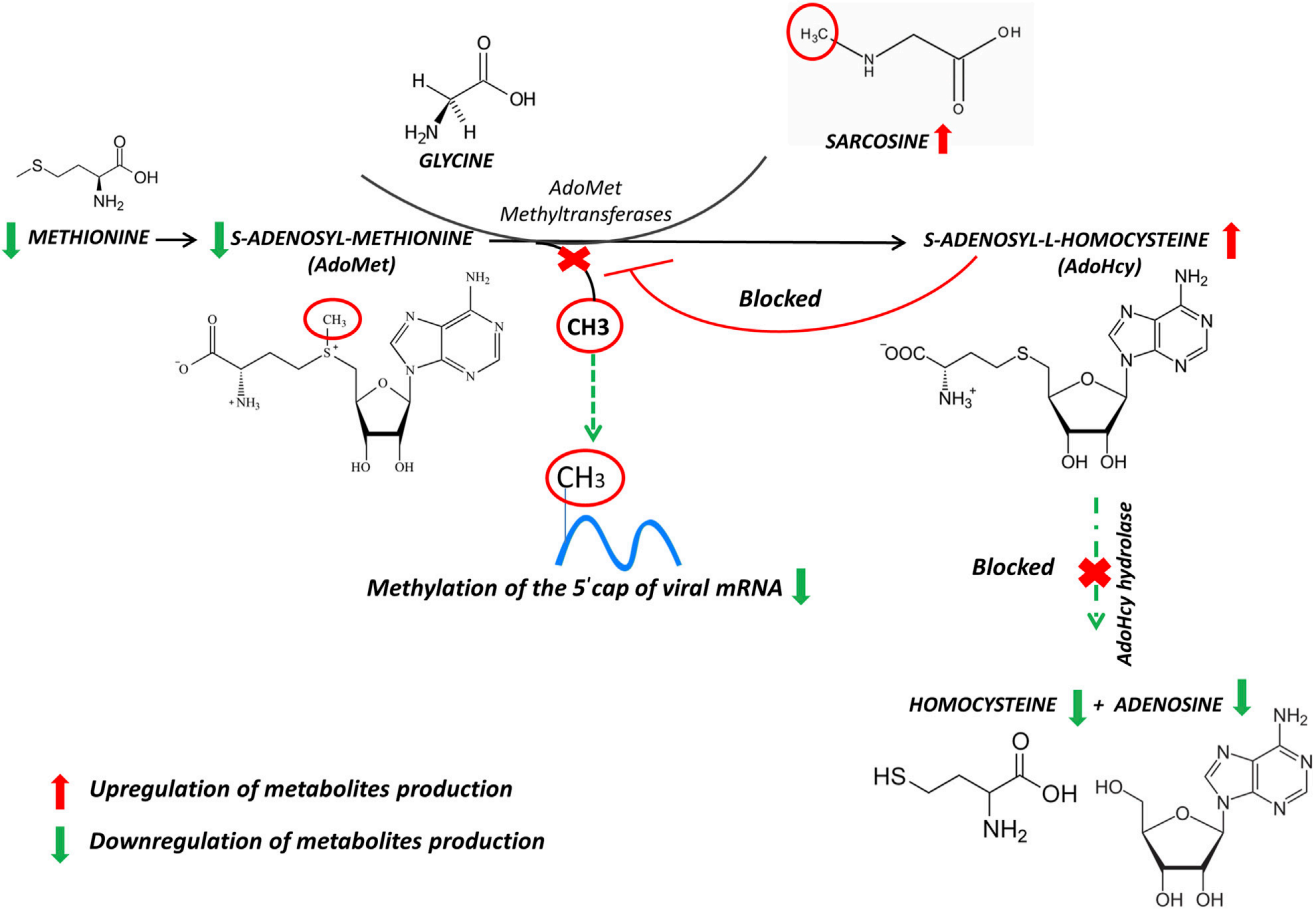
Reactions of methionine metabolism. S-adenosyl-methionine (SAM) donates its methyl group to acceptor molecules, sarcosine, generating S-adenosylhomocysteine (SAH) limiting methylation of the 5′ cap of viral messenger RNA. The arrows represent the trend of our results. Up-modulations are in red, whereas down-modulations are in green.

Metabolites were extracted from peripheral blood mononuclear cells (PBMC) cultures displaying an anti-spike-S1 IgG-memory (IgGm+; N = 17) and from PBMC without measurable IgG-memory (IgGm-; N = 24) measured by Cell-ELISA 60–90 days after SARS-Cov-2 screening. Supplementary Table S1 shows age/gender and Cell-ELISA levels for each subject. We use MetaboAnalyst 5.0 platforms to perform untargeted metabolomics and identify the most relevant metabolites altered in IgGm + subjects, and results indicate that the two different groups are well clustered, as shown in Figure 1A by the supervised Least-Squares Discriminant Analysis (PLS-DA), with red spots representing IgGm + samples and green spots IgGm-. R2 and Q2 values were thus calculated as measures of prediction accuracy (accuracy 0.82, R2 > 0.71, Q2 > 0.33; data not shown). Multivariate analysis suggests significant variations present in IgGm + subjects. The loading plot of metabolites in the analyzed samples projected into the PLS-DA model is shown in Figure 1B. The significantly-discriminated metabolites were identified using the Volcano plot analysis (Figure 1C). The univariate analysis identified a significant accumulation of specific metabolites most of which were expressed in IgGm + leukocytes. The metabolites that displayed a major alteration linked to physiological recovery from viral infections and immune activities, were arginine, sarcosine and s-adenosyl-homocysteine. The last two metabolites were involved in the methionine cycle (Figure 2).

## Discussion

The SARS-CoV-2 induces an antibody memory against the spike-S1 antigen that can be measured by *in vitro* assays in PBMC from 2 months onward after infection (Zarletti et al., 2020), and our work aimed to investigate metabolic features in these conditions. Through metabolomic analysis, the results showed that *in vitro* cultured and unstimulated PBMC display a significant up-regulation of metabolically-related metabolites sarcosine, s-adenosyl-L-homocysteine and arginine.

The S-adenosyl-methionine (AdoMet, also frequently abbreviated as SAM and SAMe) is widely known as a main biological methyl donor converted to s-adenosyl-L-homocysteine (AdoHcy), and sarcosine as an acceptor of the methyl group. The SAM to AdoHcy ratio depends also on the availability of methionine, which is converted to SAM by the enzyme methionine adenosyltransferase (MAT). AdoHcy cellular content is regulated by the enzyme AdoHcy hydrolase (AHCY), which reversibly cleaves this molecule into adenosine and homocysteine (Hcy). The thermodynamics of AHCY stimulates AdoHcy biosynthesis rather than hydrolysis, and *in vivo*, the reaction proceeds in the direction of hydrolysis only if the products, adenosine and Hcy, are rapidly removed. This is essential to prevent the accumulation of AdoHcy (Figure 2) which is a potent inhibitor of methylation reactions (Tehlivets et al., 2013).

The ratio of AdoMet to AdoHcy is frequently considered a metabolic gauge controlling *in vivo* methylation reactions, where a decrease in this ratio predicts reduced methylation capacity (Czarnecka et al., 2020). These methylations are required for the 5′-cap formation of viral mRNAs. The RNA cap has multiple roles in gene expression, including enhancement of RNA stability, splicing, nucleocytoplasmic transport, and translation initiation, necessary for viral RNA replication (Byszewska et al., 2014).

So since the methylation (capping) of viral RNA is necessary for its life cycle, a high SAM level and high methylation index are associated with the risk of lung injury in patients with COVID-19 (Kryukov et al., 2021). Metabolomic analyses revealed that SAM was significantly elevated in critical cases of COVID-19 and those with a fatal outcome as compared to control, mild and moderate cases of COVID-19 (Roberts et al., 2021; Perła-Kaján and Jakubowski, 2022).

In this respect, inhibition of the enzyme AdoHcy hydrolase can be used as a therapy against virus infection, because indirectly limits the bioavailability of SAM and methylation of the 5′ cap of viral messenger RNA as already noticed for both Ebola virus and African swine fever virus (Villalón et al., 1993; Bray et al., 2000). Moreover, it should be noted that RNA methylation in SARS Cov-2 prevents its degradation by host nucleases (Wilamowski et al., 2021) and thus might induce long-lasting effects in leukocyte metabolism. To our results, the strong increase of AdoHcy allowed us to postulate an inhibition of s-adenosyl-L-homocysteine hydrolase, and consequently, imbalance of SAM/AdoHcy ratio. Furthermore, the restricted level of methionine availability may inhibit or eliminate the replication as well as prevent proliferation of cells infected with SARS-CoV-2 (Hoffman and Han, 2020). Hence, It is also conceivable to conclude that blocking capping viral mRNAs could be an initial step to developing therapeutics that could help the antiviral therapies.

Interestingly, we also observed a strong up-regulation of arginine in IgGm + leukocytes, in addition to AdoHcy and sarcosine. Arginine is a substrate for nitric oxide (NO) production, which can induce antiviral activity against RNA viruses, such as SARS-CoV-2 (Tatum et al., 2020). The up-rugualted of the arginase 1 (Arg1), enzyme that metabolizes arginine to ornithine and urea, was associated with higher virus load in the PBMC of COVID-19 patients (Hemmat et al., 2020).

According to previous work (Fraser et al., 2020), the plasma of COVID 19 patients contains a lower amount of arginine and, as a consequence, its depletion could potentially delay and/or compromise intensive care unit recovery (Fraser et al., 2020). Concerning immune defenses, it should be noted that an increase in arginine concentration may play a role in the regulation of immune cell reactivity through the proliferation and differentiation of naive T cells to memory (Geiger et al., 2016). Arginine could preferentially enhance the proliferation of T lymphocyte subpopulations by increasing specific receptor expression and IL-2 production (Tate et al., 2008), and increasing T cell number/responses (Kim et al., 2018). Moreover, an arginine increase could also enhance affect lymphocyte proliferation and macrophage activities by providing a substrate for protein synthesis and/or precursors of polyamines or nitric oxide, being important in sustaining cellular proliferation and macrophage physiology (Geiger et al., 2016; Ji et al., 2019). Interestingly, patients with severe COVID-19 have an increased inflammatory response that depletes arginine, impairs T cell and endothelial cell function, and causes extensive pulmonary damage. Therefore, inhibition of arginase-1 and/or replenishment of arginine may be important in preventing/treating severe COVID-19 (Dean et al., 2021).

In conclusion, these preliminary results suggest that unstimulated PBMC analyzed up to 90 days after SARS-CoV-2 infection shows an increase in antiviral metabolites (sarcosine and S-adenosylhomocysteine), and a modulation of arginine metabolism, involved in innate and adaptive immunity. The analysis of these metabolites might represent a biomarker of effective and long-standing antiviral activation of PBMC. Although more studies are needed by increasing the number of samples and taking a longer time course, our work provides a basis for further analysis of protective immunity to SARS-CoV-2.

## Materials and Methods

41 subjects (23 males and 18 females, Supplementary Table S1) undergoing COVID-19 serological analysis (Centro Polispecialistico Giovanni Paolo I, Viterbo, I) were enrolled in this study from October 2020 to March 2021. All participants provided informed written consent to participate in the research project, and the study was approved by the Regional Ethical Board in Ospedale L. Spallanzani, Roma, (number 169, approval 22/07/2020) and, following the Helsinki Declaration, written informed consent was obtained from all subjects. The comorbidities from donors have been excluded on the base of the declaration released by donors when enrolled in the protocol. No specific medical analyses have been done, with the exclusion of a Covid-19 screening from declared asymptomatic donors 3 days before bleeding.

Determination of *in vitro* IgG B cell memory for spike-S1 virus protein and of specific IgG was performed by Cell-ELISA in PBMC, from all donors, employing spike-S1 coated wells from a commercial source, experimental outputs were net absorbance values (A 450 nm) with background subtracted. A cutoff value for negative samples was arbitrarily established at an OD450 value of 0.07 as previously described without modifications (Zarletti et al., 2020). Parallel cultures of PBMC employed in Cell-ELISA were incubated without stimulants at 106/ml at 37°C for 48 h in 100 ul/well of RPMI medium containing 10% FCS and antibiotics, then centrifuged at 500 xg, and pelleted cells were immediately employed for metabolomic analysis. Metabolites were extracted and analyzed by LC-MS following the protocol published in (Ambrosini et al., 2020). Each sample was added to 1,000 μl of a chloroform/methanol/water (1:3:1 ratio) solvent mixture stored at −20°C. The tubes were mixed for 30 min and subsequently centrifuged at 1,000 × *g* for 1 min at 4°C, before being transferred to −20°C for 2–8 h. The solutions were then centrifuged for 15 min at 15,000×*g* and were dried to obtain visible pellets. Finally, the dried samples were re-suspended in 0.1 ml of water, 5% formic acid, and transferred to glass autosampler vials for LC/MS analysis. Twenty microliters of extracted supernatant samples were injected into an ultrahigh-performance liquid chromatography (UHPLC) system (Ultimate 3,000, Thermo) and run on a positive mode: samples were loaded onto a Reprosil C18 column (2.0 × 150 mm, 2.5 μm-DrMaisch, Germany) for metabolite separation. For positive ion mode (+) MS analyses, a 0–100% linear gradient of solvent A (ddH2O, 0.1% formic acid) to B (acetonitrile, 0.1%formic acid) was employed over 20 min, returning to 100% A in 2 min and holding solvent A for a 1-min post time hold. Acetonitrile, formic acid, and HPLC-grade water and standards (≥98% chemical purity) were purchased from Sigma Aldrich. Chromatographic separations were made at a column temperature of 30°C and a flow rate of 0.2 ml/min. The UHPLC system was coupled online with a Q Exactive mass spectrometer (Thermo) scanning in full MS mode (2 *μ* scans) at the resolution of 70,000 in the 67–1,000 m/z range, a target of 1,106 ions, and a maximum ion injection time (IT) of 35 ms with 3.8 kV spray voltage, 40 sheath gas, and 25 auxiliary gas. Calibration was performed before each analysis against positive or negative ion mode calibration mixes (Pierce, Thermo Fisher, Rockford, IL) to ensure the error of the intact mass within the sub-ppm range. 10 μl aliquots of all samples were pooled as a quality control (QC) sample which was run every 10 samples. Replicates were exported as. mzXML files and processed through MAVEN.5.2. Multivariate Partial Least-Squares Discriminant Analysis (PLS-DA) and Univariate (Volcano plot) statistical analyses were performed on the entire metabolomics data set using the MetaboAnalyst 5.0 software. Before the analysis, raw data were normalized by sum and autoscaling to increase the importance of low-abundance ions without significant amplification of noise. This type of plot displays the fold change differences and the statistical significance for each variable. The false discovery rate (FDR) was used for controlling multiple testing (*p*-value FDR cutoff 0.05).

## Data Availability

The original contributions presented in the study are included in the article/Supplementary Material, further inquiries can be directed to the corresponding authors.

## Supplementary Material

Supplementary Table 1 Cell-ELISA absorbance (A450 nm) net values, positive values are in bold with a cutoff value of 0.07. The Cell-ELISA protocol and cutoff value areas previously described (Zarletti et al., 2020). Materials were from a commercial kit (Dia. Pro Diagnostics Bioprobes SRL, Milano, Italy. Lot 0420/5AA, Ref: COV19G.CE.192). The negatives were asymptomatic donors screened for Covid-19 at 35 and 3 days before CellELISA.
